# Cardiotoxicity Related to Immune Checkpoint Inhibitors: A Real-World Retrospective Analysis

**DOI:** 10.3389/fcvm.2022.838488

**Published:** 2022-05-31

**Authors:** Jianqing She, Hui Liu, Haoyu Wu, Gulinigaer Tuerhongjiang, Tao Zheng, Ling Bai

**Affiliations:** ^1^Cardiovascular Department, First Affiliated Hospital of Xi'an Jiaotong University, Xi'an, China; ^2^Key Laboratory of Environment and Genes Related to Diseases, Ministry of Education, Xi'an, China; ^3^Biobank, First Affiliated Hospital of Xi'an Jiaotong University, Xi'an, China

**Keywords:** cardiotoxicity, myocarditis, immune checkpoint inhibitors, PD-1, PDL-1

## Abstract

**Background:**

Cardiotoxicity related to immune checkpoint inhibitors (ICIs) is a rare but potentially lethal. In ICI-associated adverse events, evidence of cardiotoxicity and clinical predictive factors related to ICI is lacking. Here, we aim to assess the incidence and predictive factors of cardiotoxicity related to ICIs in real-world practice.

**Objective:**

We retrospectively analyzed consecutive patients who received PD-1 or PD-L1 at the First Affiliated Hospital of Xi'an Jiaotong University. Clinical characteristics and cardiac lesion markers were collected both at baseline and during longitudinal follow-up from the Biobank database. Follow-up CKMB and NT-proBNP levels and ratios were then evaluated.

**Results:**

A total of 2,304 patients with either PD-1 or PDL-1 utilization between August 2018 and April 2021 were collected. The average age was 59.44 ± 11.45 among PD-1 inhibitor utilizer and 58.97 ± 12.16 among PDL-1 inhibitor utilizer. The baseline creatine kinase isoenzyme MB (CKMB) levels were 17 ± 19 U/L in PD-1 inhibitor users and 17 ± 23 U/L in PDL-1 inhibitor users. Majority of patients were male, with advanced stage cancer, and received ICIs as second-line therapy. The longitudinal change of cardiac enzymes and NT-pro BNP were collected. Cardiac lesion as defined by three times increase of CKMB happens in only minority of patients receiving ICIs therapy. It is also identified that increased CKMB happened in PD-1 inhibitor groups, but not PDL-1 inhibitor groups.

**Conclusion:**

We evaluated the profile of cardiotoxicities caused by ICIs based on real-world experience. The cardiac lesion markers are generally unaltered, but it appears that the increased CKMB happened in PD-1 inhibitor groups, but not PDL-1 inhibitor groups.

## Introduction

Immune checkpoint inhibitors (ICIs), including programmed death molecule 1 (PD-1) and cytotoxic T cell associated antigen 4 (CTLA-4) inhibitors, have substantially improved the prognosis of a variety of cancers (1–3). Although ICIs have become the first-line treatment for a variety of solid tumors, immune related adverse events (irAEs) are increasingly recognized with the widespread utilization of these drugs (4, 5). It has been reported that ICIs can induce severe myocarditis (6, 7), but the pathogenesis, pathological mechanism and prognosis of severe myocarditis caused by ICIs are not clear.

Physiologically, PD-1 or CTLA-4 combined with its ligands can inhibit T cell activation, prevent over activation of human immune system and avoid autoimmune diseases (1–3). As important advances in tumor therapy in recent years, ICIs mainly take effect by inhibiting immune checkpoints through antibodies [such as PD-1, Programmed cell death 1 ligand 1 (PD-L1), CTLA-4, etc.], so as to restore and improve the ability of effector T lymphocytes and specifically recognize and kill tumor cells, enhancing the systemic anti-tumor immune response. While ICIs brings significant survival benefits to patients, the immunotoxicity to various organs of patients has become an unavoidable new problem in clinical treatment.

The pathophysiological mechanism of ICIs related myocarditis is not completely clear. Myocardial biopsy showed that there were a large number of T lymphocytes and macrophages infiltrating the patient's myocardial tissue, suggesting that immune inflammation is the main pathogenesis (8). It has been reported that autoantibodies such as troponin antibody, myosin antibody, and β adrenaline antibodies are also involved in the pathogenesis of ICIs related myocarditis (9, 10). The main manifestations of patients with ICIs related myocarditis were chest pain, palpitation, shortness of breath, pulmonary edema, cardiogenic shock, and arrhythmia. Cardiac markers such as troponin, CKMB, and CK are increased, and echocardiography can show left ventricular systolic dysfunction and segmental wall motion abnormalities (10). Whereas, there was no specificity in clinical manifestations, laboratory indexes and imaging changes. Endocardial biopsy was the gold standard for the diagnosis of ICIs related myocarditis. One retrospective cohort study has indicated that routine troponin monitoring may be helpful to predict the mortality of tumor patients treated with ICI in the early stage and early detection of elevated troponin is helpful to early intervention and improve the prognosis of ICI associated myocarditis (11). However, real world evidence regarding the occurrence and predictive and protective factors of ICIs related myocarditis is lacking.

In the present single-center real-world study, we retrospectively evaluated the cardiac lesion markers in patients using either 6 kinds of PD-1 inhibitors (Cindilimab, Nivolumab, Pembrolizumab, Toripalimab, Camrelizumab, Tislelizumab) or 2 kinds of PDL-1 inhibitors (Durvalumab, Atezolizumab). By analyzing the alteration of cardiac markers 1 year within the utilization of ICIs, we aimed to assess the incidence and predictive factors of cardiotoxicity related to ICIs in real-world practice.

## Methods

### Data Collection

This is a single-center retrospective study. We collected the retrospective electronic medical records from the Biobank of the First Affiliated Hospital of Xi'an Jiaotong University, which contains de-identified data derived from raw medical records. The project was approved by the Institutional Ethical Board of the First Affiliated Hospital of Xi'an Jiaotong University.

### Study Design

**Figure 1:**
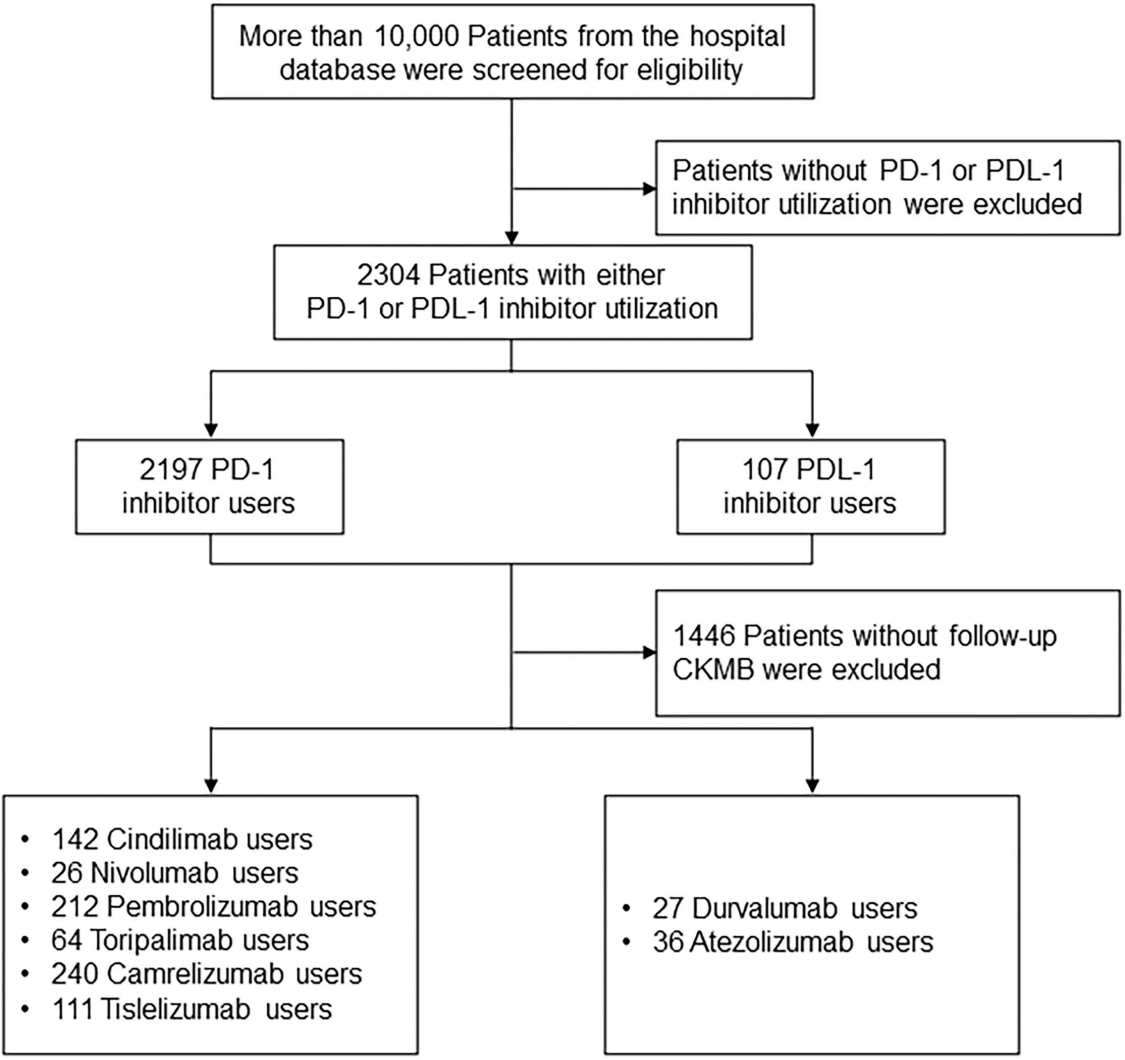
Patients' selection, screening, and follow-up.

Participants were allocated into PD-1 inhibitor (Cindilimab, Nivolumab, Pembrolizumab, Toripalimab, Camrelizumab, Tislelizumab) and PDL-1 inhibitor (Durvalumab, Atezolizumab) treatment groups. Patients were prescribed with PD-1 or PDL-1 inhibitor based on the contemporary expert consensus (12–14). The primary endpoint was the CKMB and NT-proBNP levels among patients receiving PD-1 and PDL-1 treatment during drug initiation, and 1 month and 6 months after the treatment. More than 10,000 patients diagnosed with cancer from the hospital database were screened for eligibility. Patients without PD-1 or PDL-1 inhibitor utilization were excluded, resulting in 2,304 Patients with either PD-1 or PDL-1 utilization. The cohort entry date was the date of the first prescription of ICIs. A total of 1,446 Patients without follow-up CKMB were excluded, and 795 PD-1 inhibitor users (142 Cindilimab, 26 Nivolumab, 212 Pembrolizumab, 64 Toripalimab, 240 Camrelizumab, 111 Tislelizumab), as well as 63 PDL-1 inhibitor users (27 Durvalumab, 36 Atezolizumab) were analyzed in the follow-up analysis (Figure 1).

### Data Collection and Cardiac Biomarker Measurement

Demographic and epidemiological data including age, sex, and disease history were collected upon admission. Serum samples were collected from the patients upon admission, and N-terminal pro-B-type natriuretic peptide (NT-proBNP), and cardiac lesion markers including cTnI, Myo, CK, CKMB, HBDB, and LDH were tested by the laboratory department.

### Statistical Analysis

All statistical analyses were performed by SPSS for Windows 25.0 (SPSS Inc. Chicago, IL, USA). Data were presented as frequencies and percentages for categorical variables and mean ± SD for continuous variables, unless otherwise indicated. Simple *t*-test was used to compare continuous variables which are in normal distribution. Mann-Whitney U test was used to compare continuous variables which do not conform to the normal distribution. One-way ANOVA was used to compare continuous variables or χ2 test for categorical variables. Two-sided *P* values of < 0.05 were considered to indicate statistical significance.

### Role of the Funding Source

The funding agencies did not participate in study design, data analysis, or writing of the report. The corresponding authors had full access to all aspects of the study to ensure the accuracy or integrity of any part of the work. The final version was approved by all authors.

## Results

### Study Cohort and Baseline Characteristics

A total of 2,304 patients with either PD-1 or PDL-1 utilization between August 2018 and April 2021 were collected in the present study. Characteristics of the cohorts utilizing PD-1 and PDL-1 were presented in Table 1. The average age was 59.44 ± 11.45 among PD-1 inhibitor utilizer and 58.97 ± 12.16 among PDL-1 inhibitor utilizer. The baseline Creatine kinase isoenzyme MB (CKMB) levels were 17 ± 19 U/L in PD-1 inhibitor users and 17 ± 23 U/L in PDL-1 inhibitor users. No significant difference was observed in the baseline sex, age, lactic dehydrogenase (LDH), creatine kinase (CK), troponin T (cTNT); troponin I (cTNI); N-terminal B-type Natriuretic Peptide (NT-proBNP) levels.

**Table 1:** Baseline information of patients receiving PD-1 or PDL-1 inhibitor.

	**PD-1 inhibitor**	**PDL-1 inhibitor**	***P* value**
Number	2,159	145	
Sex (Female%)	31.50%	26.21%	
Age	59.44 ± 11.45	58.97 ± 12.16	ns
**Cancer type**			
Lung (%)	44.93%	80.14%	
Liver (%)	21.82%	8.22%	
Intestine (%)	26.45%	2.74%	
Bone (%)	5.47%	6.16%	
Others (%)	1.34%	2.74%	
LDH	262.85 ± 246.69	252.16 ± 167.42	ns
HMDB	208.28 ± 155.13	199.35 ± 110.89	ns
CK	17.32 ± 15.7	17.48 ± 22.65	ns
CKMB	17 ± 19	17 ± 23	ns
cTNT	0.02 ± 0.04	0.01 ± 0.02	ns
cTNI	10.98 ± 41.52	21.96 ± 65.23	ns
NT-proBNP (Log2 transform)	6.73 ± 2.07	6.18 ± 2.04	ns

*LDH, Lactic Dehydrogenase; CK, Creatine kinase; CKMB, Creatine kinase isoenzyme MB; cTNT, troponin T; cTNI, troponin I; NT-proBNP, N-terminal B-type Natriuretic Peptide; ns, not significant*.

### CKMB and NT-ProBNP Levels Among Patients Utilizing PD-1 or PDL-1 Inhibitor

The CKMB levels during treatment initiation and 1 and 6 months after the treatment are shown in Table 2. No statistically significant difference was observed from treatment initiation until 6-month follow-up among all kinds of PD-1 and PDL-1 inhibitors (Table 2). Similarly, the Log2 transformed NT-proBNP levels were not significantly altered during the drug initiation, 1 month and 6 months after the treatment both in PD-1 and PDL-1 inhibitors utilizers (Table 3). These results indicate that the PD-l and PDL-1 are clinically safe treatments, and the myocardial lesion occurs only in a very small number of patients.

**Table 2:** CKMB levels among paients utilizing PD-1 or PDL-1 inhibitor.

	**Initiation (U/L)**	**1 Month** **(U/L)**	**6 Month (U/L)**	***P* value**	**Increased CKMB (%)**	***P* value (χ2 test)**
**PD-1 inhibitor**						
Cindilimab	17 ± 13	19 ± 23	19 ± 28	ns	2.11%	ns
Nivolumab	23 ± 32	24 ± 38	38 ± 91	ns	7.69%	
Pembrolizumab	16 ± 11	16 ± 12	16 ± 14	ns	1.89%	
Toripalimab	16 ± 13	17 ± 13	16 ± 13	ns	1.56%	
Camrelizumab	19 ± 15	19 ± 17	18 ± 16	ns	1.25%	
Tislelizumab	17 ± 22	15 ± 16	15 ± 14	ns	4.50%	
**PDL-1 inhibitor**						
Durvalumab	17 ± 7	16 ± 13	14 ± 10	ns	0.00%	ns
Atezolizumab	19 ± 29	16 ± 17	16 ± 28	ns	0.00%	

*ns, not significant*.

**Table 3:** NT-proBNP levels among paients utilizing PD-1 or PDL-1 inhibitor.

	**Initiation (pg/mL)**	**1 Month** **(pg/mL)**	**6 Month (pg/mL)**	***P* value**
**PD-1 inhibitor**				
Cindilimab	6.80 ± 2.04	6.49 ± 1.95	6.59 ± 1.93	ns
Nivolumab	8.23 ± 1.59	7.87 ± 1.70	8.51 ± 1.75	ns
Pembrolizumab	6.70 ± 2.17	6.70 ± 1.91	6.51 ± 1.91	ns
Toripalimab	6.21 ± 2.12	6.20 ± 2.04	6.37 ± 2.00	ns
Camrelizumab	6.58 ± 1.80	6.52 ± 1.74	6.49 ± 1.77	ns
Tislelizumab	6.98 ± 2.36	6.84 ± 2.17	6.69 ± 2.24	ns
**PDL-1 inhibitor**				
Durvalumab	6.17 ± 2.21	5.89 ± 2.37	6.00 ± 2.29	ns
Atezolizumab	6.19 ± 1.70	6.75 ± 1.65	6.96 ± 1.77	ns

*ns, not significant*.

### Percentage of Patients With CKMB Increase and Relative CKMB Levels in Patients Utilizing PD-1 and PDL-1 Inhibitors

**Figure 2:**
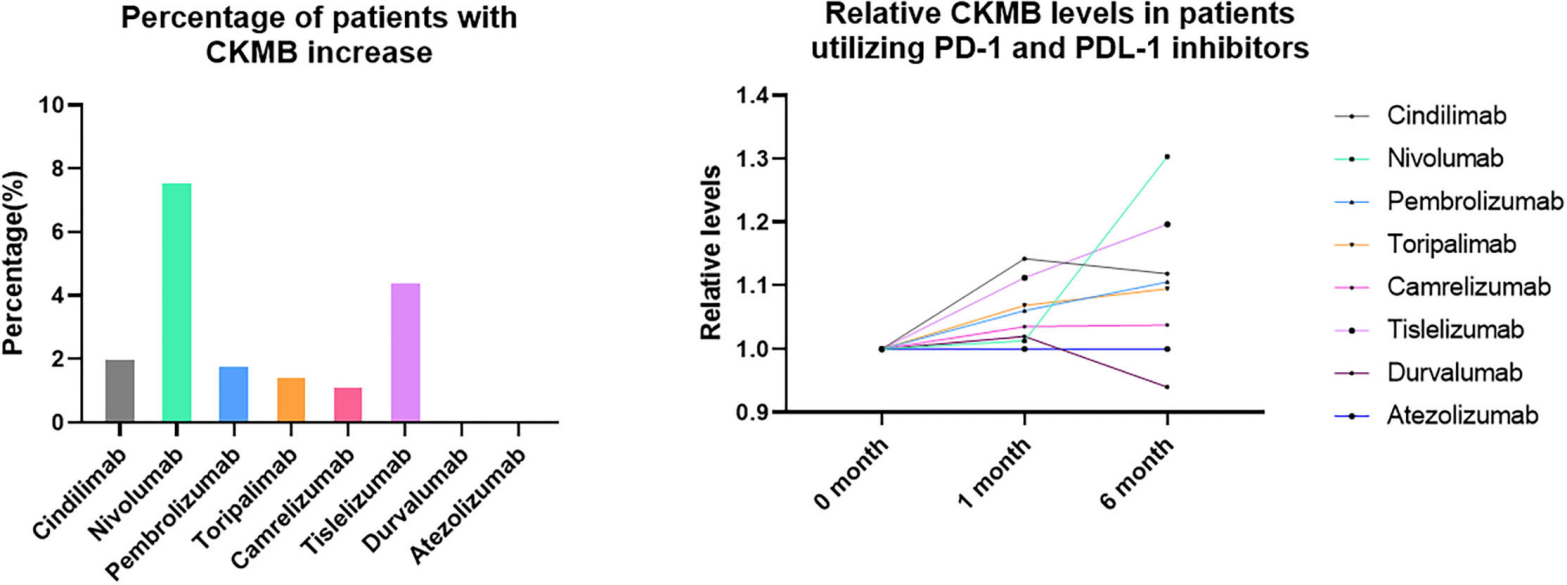
Percentage of patients with CKMB increase and relative CKMB levels in patients utilizing PD-1 and PDL-1 inhibitors.

Although no significant differences were observed with regard to the CKMB and NT-proBNP levels during the follow-up time points and in different treatment groups, abnormal increase of CKMB were noticed in the PD-1 inhibitor group. When we define the increase of CKMB levels as more than 3 times higher than baseline levels (15) either during 1 month and 6-month follow-up, 2.11% patients had increased CKMB in Cindilimab group; 7.69% in Nivolumab, 1.89% in Pembrolizumab, 1.56% in Toripalimab, 1.25% in Camrelizumab, and 4.50% in Tislelizumab, respectively. On the contrary, no patients with increased cardiac enzymes were observed in PDL-1 inhibitor utilizers, both in Durvalumab and Atezolizumab group. The percentage of patients with CKMB increase and relative CKMB levels in patients utilizing PD-1 and PDL-1 inhibitors were shown in Figure 2.

## Discussion

In the present real-world study to evaluate the cardiac lesion marker in patients using either PD-1 inhibitors or PDL-1 inhibitors, we found that the cardiac lesion markers are generally unaltered both in PD-1 and PDL-1 inhibitor groups, further confirming that cardiac lesion happens in only minority of patients receiving ICIs therapy. It is also identified that increased CKMB happened in PD-1 inhibitor groups, but not PDL-1 inhibitor groups.

With the increased use of immune checkpoint inhibitors (ICIs), the associated adverse events are becoming common (16). Blocking immune checkpoints to restore antitumor immune response may also break immune tolerance to self-antigens and induce specific immune-related adverse events (17). Treatment with ICIs can lead to severe and disabling inflammatory cardiovascular adverse-events soon after the commencement of therapy (18, 19). Specifically, myocarditis caused by ICIs represent <1% of immune-related adverse events. It is a potentially fatal condition associated with a mortality rate of 42% (20). The keys to the diagnosis in this case are the previous history of PD-L1 utilization, elevated biomarkers suggesting cardiac damage and decreased left ventricular ejection fraction (21, 22). Our present study focuses on the longitudinal change of CKMB and NT-proBNP. It is suggested that although cardiac lesion does occur among patients receiving ICIs, the incidence is generally low, with no significant difference among different kinds of ICIs.

It is worthy of attention that the increased CKMB happened in PD-1 inhibitor groups, but not in PDL-1 inhibitor groups. Due to the discrepancy of the sample numbers, no statistical significance is observed between the two treatment groups. However, it brings to our attention that no cases with significant increase of CKMB are identified in PDL-1 group. This is in accordance with the previous report that PD-L1 as monotherapy for lung cancer has been rarely reported to cause acute myocarditis (23). Mechanistically, PD-1 is an inhibitory receptor expressed on activated T cells, B cells, macrophages, regulatory T cells, and natural killer (NK) cells. PDL-1 is abundantly expressed on the surface of tumor cells, reducing T cell activation, and antigen-specific T cell immune response, thus bypassing immune surveillance (23). As a result, theoretically, patients treated with PD-1 inhibitors are more likely to be interfered with the function of self-immune cells than PDL-1 inhibitors, resulting in damage to their own organs. The above hypotheses need to be further verified and clarified by larger clinical trials and mechanism studies.

Our findings should be interpreted in the context of several potential limitations. As this is a retrospective study, the follow-up data of echocardiography and New York Heart Association functional class are not complete, although these variables have been shown to contribute to the diagnostic and prognostic information in patients with myocarditis. In addition, the clinical diagnoses of ICI related myocarditis should include patients' medical history, clinical presentation, echocardiography presentation as well as laboratory markers including cardiac enzymes, so the present study provided relatively limited information by focusing only on the cardiac enzymes and NT-proBNP alteration. Next, different types of cancer and other tumor treatments might affect the myocardial injury markers may among these patients. At last, the incomplete information about the patients' medication compliance might also affect the endpoints. As this is a single-center retrospective study, the follow-up results are not broadly representative.

## Conclusion

This retrospective study based on real-world data suggests that the cardiac lesion markers are generally unaltered both in PD-1 and PDL-1 inhibitor groups. It also appears that increased CKMB happened in PD-1 inhibitor groups, but not PDL-1 inhibitor groups. Myocarditis as an adverse event has low incidence and high mortality, which deserves immediate recognition. Physicians should be aware of these infrequent, but potentially fatal toxicities associated with ICIs as their therapeutic use becomes widespread.

## Data Availability Statement

The raw data supporting the conclusions of this article will be made available by the authors, without undue reservation.

## Ethics Statement

The studies involving human participants were reviewed and approved by the Institutional Ethical Board of the First Affiliated Hospital of Xi'an Jiaotong University. The patients/participants provided their written informed consent to participate in this study.

## Author Contributions

JS, HL, and LB participated in the design of the study. JS, HW, and HL collected the patients' data and did the follow-up. HW and GT performed the statistical analysis. GT and TZ finished the patients' follow-up. JS and LB drafted the manuscript. All authors approved the final manuscript.

## Funding

This study was supported by National Natural Science Foundation of China (Nos. 81800390 and 82170464), National Key R&D Program of China (2019YFA0802300 and 2018YFC1311505), Key Research and Development Program of Shaanxi (Nos. 2020KW-049, 2017SF-085, S2020-YF-GHMS-0014, 2017SF-085, and 2020GXLH-Y-015), Central University Basic Science Foundation of China (119132971000056), and the Clinical Research Award of the First Affiliated Hospital of Xi'an Jiaotong University, China (Nos. XJTU1AF-CRF-2018-025 and XJTU1AF-CRF-2017-006).

## Conflict of Interest

The authors declare that the research was conducted in the absence of any commercial or financial relationships that could be construed as a potential conflict of interest.

## Publisher's Note

All claims expressed in this article are solely those of the authors and do not necessarily represent those of their affiliated organizations, or those of the publisher, the editors and the reviewers. Any product that may be evaluated in this article, or claim that may be made by its manufacturer, is not guaranteed or endorsed by the publisher.

## Abbreviations

ICIs: Immune Checkpoint Inhibitors
PD-1: Programmed Death Molecule 1
PD-L1: Programmed Cell Death 1 Ligand 1
CTLA-4: Cytotoxic T cell Associated Antigen 4
irAEs: Immune Related Adverse Events
LDH: Lactic Dehydrogenase
CK: Creatine kinase
CKMB: Creatine kinase isoenzyme MB
cTNT: troponin T
cTNI: troponin I
NT-proBNP: N-terminal B-type Natriuretic Peptide.
